# Efficient CRISPR-Cas13d-Based Antiviral Strategy to Combat SARS-CoV-2

**DOI:** 10.3390/v15030686

**Published:** 2023-03-06

**Authors:** Mouraya Hussein, Zaria Andrade dos Ramos, Monique A. Vink, Pascal Kroon, Zhenghao Yu, Luis Enjuanes, Sonia Zuñiga, Ben Berkhout, Elena Herrera-Carrillo

**Affiliations:** 1 Laboratory of Experimental Virology, Department of Medical Microbiology, Amsterdam UMC, Academic Medical Center, University of Amsterdam, 1105 AZ Amsterdam, The Netherlands; 2 Department of Molecular and Cell Biology, National Center of Biotechnology (CNB-CSIC), Campus Universidad Autónoma de Madrid, 28049 Madrid, Spain

**Keywords:** COVID-19, SARS-CoV-2 genome, CRISPR-Cas13d, RNA

## Abstract

The current SARS-CoV-2 pandemic forms a major global health burden. Although protective vaccines are available, concerns remain as new virus variants continue to appear. CRISPR-based gene-editing approaches offer an attractive therapeutic strategy as the CRISPR-RNA (crRNA) can be adjusted rapidly to accommodate a new viral genome sequence. This study aimed at using the RNA-targeting CRISPR-Cas13d system to attack highly conserved sequences in the viral RNA genome, thereby preparing for future zoonotic outbreaks of other coronaviruses. We designed 29 crRNAs targeting highly conserved sequences along the complete SARS-CoV-2 genome. Several crRNAs demonstrated efficient silencing of a reporter with the matching viral target sequence and efficient inhibition of a SARS-CoV-2 replicon. The crRNAs that suppress SARS-CoV-2 were also able to suppress SARS-CoV, thus demonstrating the breadth of this antiviral strategy. Strikingly, we observed that only crRNAs directed against the plus-genomic RNA demonstrated antiviral activity in the replicon assay, in contrast to those that bind the minus-genomic RNA, the replication intermediate. These results point to a major difference in the vulnerability and biology of the +RNA versus −RNA strands of the SARS-CoV-2 genome and provide important insights for the design of RNA-targeting antivirals.

## 1. Introduction

The outbreak of Coronavirus Disease 2019 (COVID-19) at the end of 2019 has created a profound global health burden with pandemic-scale numbers of infected and fatal cases [1,2,3]. COVID-19 is caused by the severe acute respiratory syndrome coronavirus-2 (SARS-CoV-2) [4,5]. As of August 2022, the World Health Organization has confirmed some 572 million human cases worldwide and 6.4 million (1.1%) deaths. The pandemic spurred research towards the development of protective vaccines at an unprecedented pace, resulting in the approval of four different vaccines by the U.S. Food and Drug Administration (FDA) and the European Medicines Agency (EMA) [6,7,8,9,10,11]. However, new SARS-CoV-2 variants continue to appear and some are designated variants of concern (VOC) that may escape from these vaccines, e.g., the recent Omicron lineage [12,13]. This means that it remains prudent to develop alternative antiviral strategies in parallel. This therapeutic track should ideally consider the genetic variation among SARS-CoV-2 strains and thus prepare for a future zoonotic outbreak of an unrelated or yet unknown coronavirus [14,15,16]. The FDA has approved different antiviral drugs for COVID-19 treatment (Remdesivir and Baricitinib) and authorized others for emergency use (Molnupiravir and Paxlovid). However, there is currently no antiviral treatment that directly attacks the viral RNA genome [17,18]. To this end, the innate RNA interference (RNAi) mechanism was used to develop a novel therapeutic option against the virus [19,20,21]. We and others proposed an alternative antiviral approach based on the CRISPR genome editing tool for the specific recognition and degradation of the SARS-CoV-2 RNA genome [22,23,24].

The current COVID-19 pandemic, which started in 2019, represents the third zoonotic outbreak of a pathogenic coronavirus (SARS-CoV-2) in a relatively short timeframe after MERS-CoV in 2012 [25] and SARS-CoV in 2003 [26,27]. Moreover, the continuous emergence of new virus variants in the current SARS-CoV-2 pandemic is urging us to design antiviral strategies that are not only robust and specific, but ideally also capable of covering the genetic variation present in the virus variants. The clustered regularly interspaced short palindromic repeat (CRISPR)-Cas toolbox provides a relatively straightforward platform to design antivirals that target viral RNA or DNA in a sequence-specific manner [28,29,30]. We opted to target the SARS-CoV-2 RNA genome with the CRISPR-Cas13 system because it selectively cleaves RNAs that are complementary to the designed CRISPR-associated RNA (crRNA) and because it theoretically allows for an RNA attack anywhere in the cell (nucleus, cytosol and other subcellular compartments such as double-membrane vesicles (DMVs) that support viral RNA synthesis).

**Figure 1:**
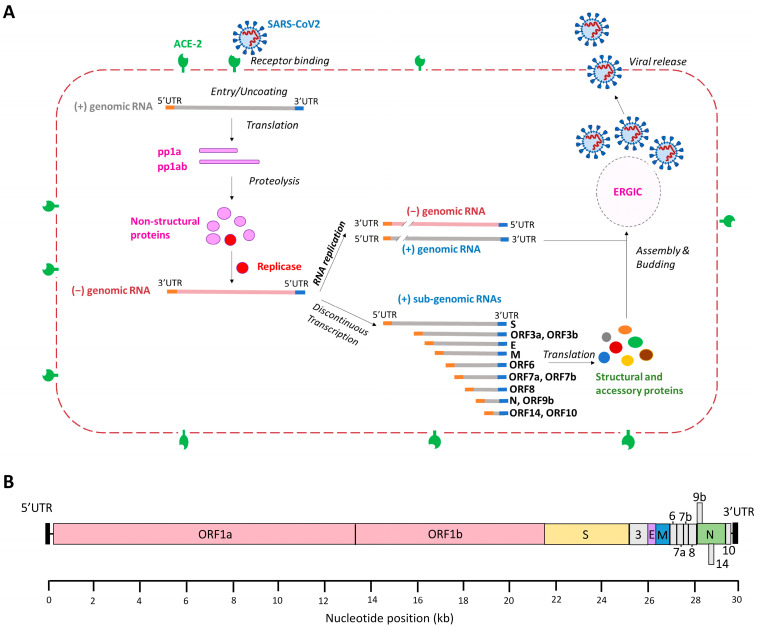
Replication cycle of SARS-CoV-2 and genome organization. (**A**) The SARS-CoV-2 replication cycle. Binding of the viral S protein to the ACE-2 receptor triggers SARS-CoV-2 infection and the plus genomic RNA (+gRNA) is translated into polyprotein (pp)1a and pp1ab. These proteins are proteolytically cleaved to generate 16 non-structural proteins (nsps), including some that form the replication/transcription complex that drives the synthesis of minus genomic RNA (−gRNA). The −gRNA is converted into genomic +gRNA that is packaged into new virion particles. Discontinuous transcription generates a set of 3′ co-terminal sub-genomic mRNAs (sgmRNAs) with identical 5′-leader and 3′-trailer ends. The sgmRNAs are translated into structural and accessory proteins that are required for the assembly of infectious virions, which takes place at the ER-Golgi intermediate compartment (ERGIC). Nascent virions are released from the cell via exocytosis. (**B**) The SARS-CoV-2 RNA genome. Human coronaviruses contain the largest viral genome (27–31 kb) among the RNA viruses and they share a similar genome organization. At the 5′-terminus two large overlapping open reading frames (ORFs), ORF 1a and ORF 1b, encode non-structural proteins and the 3′-terminal ORFs encode four structural proteins: spike (S), envelope (E), membrane (M) and nucleocapsid (N). The SARS-CoV-2 genome encodes eight accessory proteins: 3, 6, 7a, 7b, 8, 9, 14 and 10. Accessory proteins are indicated in grey.

Four different RNA-targeting Cas13 orthologues have been described (Cas13a, b, c and d) [31]. We selected Cas13d as it combines superior activity with a small size, which is beneficial for gene delivery by means of viral vectors [32,33,34]. Moreover, Cas13d lacks sequence constraints besides the crRNA target sequence, meaning that in principle any viral RNA sequence can be attacked. CRISPR-Cas13d uses a customizable ~23-nucleotide (nt) spacer to direct the endonuclease to specific RNA molecules for targeted RNA degradation [31,35,36,37]. We propose to attack either the SARS-CoV-2 +RNA or –RNA strand. Once a host cell is infected by SARS-CoV-2, the plus-genomic RNA (+gRNA) genome is released into the cytoplasm to act as mRNA for translation of the polyproteins pp1a and pp1ab (Figure 1A). The polyproteins are proteolytically processed into the different non-structural proteins (nsps) with different functions. Several viral proteins constitute the replication/transcription complex (RTC) that associates with DMVs in the perinuclear regions. That is where a full-length complementary minus-genomic RNA (−gRNA) is synthetized, which is subsequently used as template for the production of new +gRNA molecules. Additionally, a set of 3′ co-terminal sub-genomic mRNAs (sgmRNAs) with an identical 5′ leader sequence are produced from intermediate minus sub-genomic RNAs (sgRNAs) via an orchestrated process of discontinuous transcription (Figure 1A). Thus, the +gRNA and all sgmRNAs share an identical leader sequence in the 5′UTR (untranslated region) and an identical 3′UTR, making these terminal domains ideal targets for an antiviral attack on all sgmRNAs. In theory, both the viral + and −RNA strands can be attacked, but the latter may be an easier target because of the much lower copy number in the infected cell.

We set out to find optimal target sequences in the SARS-CoV-2 RNA genome for Cas13d (Figure 1B). We selected the most conserved targets [33,34] that cover all VOCs known to date and SARS-CoV [26,38]. An additional rationale for targeting conserved sequences is that viral escape will be more difficult at sites that encode important viral sequences, and escape by mutation will likely cause a drop in replication capacity [39]. For instance, the highly conserved ribosomal frameshift site has been an attractive target for anti-SARS-CoV-2 strategies, which resulted in the inhibition of frameshifting during RNA translation and virus replication [40,41,42]. Based on the alignment of the complete viral genomes of SARS-CoV and the current SARS-CoV-2 strains, we composed a CRISPR platform comprised of two sets of 29 crRNAs targeting highly conserved regions on the + or −RNA strand. We aimed at obtaining crRNAs with a broad antiviral potential against multiple human coronaviruses and at gaining mechanistic insights into factors that could affect the target RNA vulnerability based on the specific biology of human coronaviruses.

## 2. Materials and Methods

### 2.1. Design of crRNAs for a Cas13d-Mediated Attack on SARS-CoV-2

**Figure 2:**
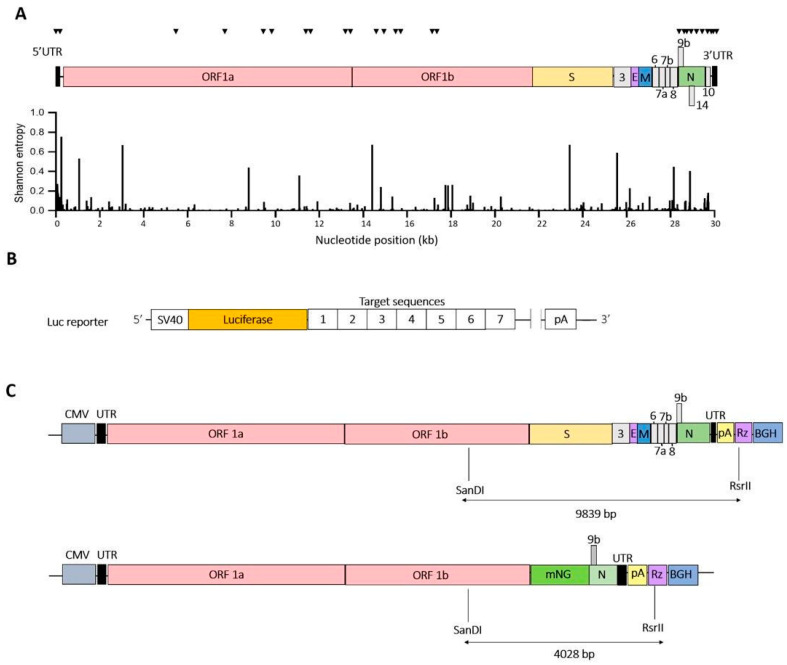
Targeting of SARS-CoV-2 RNA with CRISPR/Cas13d. (**A**) SARS-CoV-2 RNA genome diversity and the crRNA targets. Distribution of crRNAs along the SARS-CoV-2 RNA genome (MN908947). The Shannon entropy along the RNA genome varies from 0 to 1, where lower genetic diversity gives values closer to zero.The position of the crRNAs targeting conserved RNA sequences of SARS-CoV-2 is indicated with black triangles. The SARS-CoV-2 RNA genome diversity is shown by plotting Shannon entropy along the whole genome. The values vary between 0 and 1, where more conserved sequences give lower values (closer to 0); (**B**) Luciferase multitarget reporter construct. To measure the efficiency of the designed crRNAs, a pGL3 control plasmid-based multi-target luciferase reporter construct was designed with SARS-CoV-2 conserved target sequences cloned downstream of the firefly luciferase gene. Two constructs were designed: one with the + and one with the −RNA target sequences. SV40: simian virus 40 promoter, pA: polyA sequence; (**C**) Engineering a SARS-CoV-2 replicon. Scheme of the SARS-CoV-2 cDNA cloned in a BAC (upper panel) and the SARS-CoV-2 replicon (lower panel). The letters indicate the viral genes: S, spike; E, envelope; M, membrane; N, nucleocapsid; or the reporter gene mNeonGreen (mNG). UTR, untranslated regions; CMV, cytomegalovirus promoter; pA, polyA sequence; Rz, hepatitis delta virus ribozyme; BGH, bovine growth hormone polyadenylation and termination signals.

Cas13d employs a customizable crRNA that directs the RNA endonuclease to a specific sequence on the target RNA for cleavage and subsequent degradation [36]. We first ran a computational model (R script) developed by Wessels et al. along the SARS-CoV-2 genome of the Wuhan Hu-1 reference strain (MN908947) to identify 23 nt crRNAs with a high predicted efficacy [43] (Figure 2A). Then, we selected conserved target sequences by aligning the reference SARS-CoV-2 genome with 31,576 viral genome sequences, including the VOCs Alpha, Beta, Gamma, Delta and Omicron as well as the de-escalated variants Lambda and Mu (https://www.gisaid.org/ (accessed on 8 December 2021, Table S1) [22]. In addition, SARS-CoV-2 sequences were aligned with the complete genomes of SARS-CoV (260 strains). The Shannon entropy of the aligned sequences was calculated as a measure of the genetic variability per nucleotide position and plotted with Bioedit in Figure 2A [44,45]. The Shannon entropy ranges from 0 to 1, where values close to zero represent low genetic diversity (Table S2). Based on the generated list of crRNAs from the R script and the conserved target regions selected from the multiple whole-genome alignments, a set of 29 crRNAs was selected with no predicted off-target effect based on alignment with the complete transcriptome of human cells (Table 1). The selected target sites on the SARS-CoV-2 RNA genome are indicated in Figure 2A. We selected predicted weak and strong crRNAs to validate the power of the on-target prediction tool developed by Wessels et al. (Table 1).

Two sets of crRNAs were designed against either the + or the -RNA strand of SARS-CoV-2. We selected targets in the 5′UTR and 3′UTR as the genomic and sub-genomic RNAs share common sequences in these termini. We included crRNAs targeting the transcription-regulating sequence (TRS) present the 5′-leader sequence (TRS-Leader) and at the gene junctions (TRS-B), which we named 5′UTR-1 and 5′UTR-2, as these are critical for the key process of discontinuous transcription and present in the +gRNA as well as sgmRNAs [43,44]. Another highly conserved element at the 3′ end of the coronavirus genome is the s2m motif, which was recently proposed as a promising target for antivirals [45,46]. We also selected target sequences that encode critical enzymes involved in virus replication and transcription, e.g., the helicase and RNA-dependent RNA polymerase (RdRp) (Table 1).

### 2.2. Plasmid Construction

The expression plasmid pLentiRNACRISPR_005-hU6-DR_BsmBI-EFS-RfxCas13d-NLS-2A-Puro-WPRE (Addgene, #138147) expresses the RfxCas13d endonuclease in mammalian cells. This construct was kindly donated by Neville Sanjana [47]. The EF-1a core promoter was used for expression of the CaS13d nuclease and the U6 polymerase-3 promoter was used for crRNA expression. Oligonucleotides encoding SARS-CoV-2-targeting crRNAs and non-targeting control crRNA (NC) were ligated into the Esp3i site of the pLentiRNACRISPR_005-hU6-DR_BsmBI-EFS-RfxCas13d-NLS-2A-Puro-WPRE vector. All crRNAs are listed in Table S3, including a non-targeting control crRNA that does not target any sequence in the human and coronavirus genomes. All constructs were sequence-verified using the BigDye Terminator Cycle Sequencing kit (ABI). For sequencing of crRNA constructs a denaturation temperature of 98 °C was used in the presence of 1M Betaine to disrupt structures in the DNA template. The luciferase reporter plasmid was constructed by the insertion of a 250 bp fragment into the Xbal site of the pGL3-control plasmid (Promega; GenBank: U47296.2) to create EcoRI and PstI sites (Figure 2B) [48]. DNA fragments encoding the luciferase reporter with multiple CRISPR targets were synthesized by Integrated DNA Technologies (IDT, Coralville, Iowa) and cloned, one for +gRNA as well as sgmRNAs targets and one for −gRNA as well as sgRNAs targets. In the context of our experiments, we will refer to +gRNA and sgmRNAs as +RNA targets and –gRNA and sgRNAs as –RNA targets.

The SARS-CoV-2 infectious cDNA clone based on the SARS-CoV-2 Wuhan Hu-1 strain (MN908947) was assembled in a bacterial artificial chromosome (BAC) using an approach described previously [49]. To generate a SARS-CoV-2 replicon that can be used in a BSL-2 level laboratory, the structural and genus-specific genes were removed and replaced by a reporter gene (Figure 2C). The nucleocapsid (N) gene was maintained as it is required for efficient RNA synthesis [50]. A 4028 bp fragment flanked by SanDI and RsrII restriction sites was chemically synthesized (IDT, Coralville, Iowa). This DNA fragment encodes part of the SARS-CoV-2 genome (nt 20085 to 21555 corresponding to ORF1b), the mNeonGreen (mNG) reporter gene under control of the transcription regulating sequence (TRS) of the membrane (M) gene (TRS-M, nt 26431 to 26522), and another SARS-CoV-2 fragment (nt 28177 to 29533 encoding the N protein and its TRS). The synthetic fragment was inserted into the SanDI and RsrII restriction sites of pBAC-SARS-CoV2 to generate the replicon pBAC-SARSCoV-2-mNG (Figure 2C). The SARS-CoV replicon pBAC-SARS-CoV-REP was previously generated for the SARS-CoV Urbani strain (AY278741) [51]. pBAC-SARS-CoV-REP encodes a modified version of *Montastraea cavernosa* Green Fluorescent Protein (hmGFP) (Figure 2C).

### 2.3. Cell Culture and DNA Transfection

Human embryonic kidney (HEK) 293T cells were cultured in DMEM (Life Technologies, Invitrogen, Carlsbad, CA, USA) supplemented with 10% fetal calf serum (FCS), penicillin (100 U/mL), streptomycin (100 mg/mL) and 1% L-glutamine. The cells were cultured in a humidified chamber at 37 °C and 5% CO_2_.

For luciferase assays, HEK293T cells were seeded one day before transfection in 24-well plates at a density of 1.4 × 10^5^ cells per well in 0.5 mL media. Cells were transfected with 100 ng of the firefly luciferase expression plasmid, 1 ng of Renilla luciferase expression plasmid (pRL) and 300 ng of CRISPR-Cas13d/crRNA vector using Lipofectamine 2000 reagent (Invitrogen) according to the manufacturer’s instructions [52]. For the titration transfection, cells were transfected with 100 ng of firefly luciferase expression plasmid, 1 ng of pRL and 75, 150 or 300 ng of CRISPR-Cas13d/crRNA. pBS plasmid was supplemented to reach an equal amount of DNA (300 ng) per sample. The knockdown efficiency was quantified by measuring the firefly luciferase (Luc) fluorescence signal at two days post-transfection according to the manufacturer’s instructions [53]. A non-targeting crRNA served as negative control (NC), and the luciferase level scored for this construct was set at 100%. We performed three independent transfections, each in duplicate. The ratio between firefly and Renilla luciferase activity was used for normalization of the experimental variation such as differences in the transfection efficiency. The luciferase data were subsequently corrected for between-session variation using Factor Correction v10.5 [54]. The resulting six values were used to calculate the standard deviation shown as error bars. Data were analyzed with the Prism software (GraphPad Prism 9.1.0). One-way ANOVA was used for all statistical analyses: * *p* ≤ 0.05; ** *p* ≤ 0.01; *** *p* ≤ 0.001; **** *p* ≤ 0.0001.

### 2.4. SARS-CoV-2 Replicon Assay

HEK293T cells were plated a day before transfection in 24-well plates at a density of 1.4 × 10^5^ cells per well in 0.5 mL media. For transfection, we used 300 ng vector encoding Cas13d and crRNA, 1.43 μg pBAC-SARS-CoV-REP or pBAC-SARS-CoV-2-mNG, and Lipofectamine 2000 according to the manufacturer’s instructions (Invitrogen) [52]. At two days post-transfection, cells were washed twice with FACS (Fluorescence Activated Cell Sorting) buffer (PBS supplemented with 5% FCS and 2mM EDTA). The number of GFP- or mNeonGreen-positive cells was measured with flow cytometry and analyzed using FlowJo™ v10.7 [50,55]. Three independent experiments were performed in duplicate. The resulting six values were factor-corrected for between-session variation and used to calculate the standard deviation shown as error bars. Data were analyzed with the Prism software (GraphPad Prism 9.1.0). One-way ANOVA was used for all statistical analyses: * *p* ≤ 0.05; ** *p* ≤ 0.01; *** *p* ≤ 0.001; **** *p* ≤ 0.0001.

## 3. Results

### 3.1. Design of crRNAs against Highly Conserved SARS-CoV-2 RNA Sequences

We ran the R script algorithm developed by Wessels et al. [47] along the MN908947 reference genome of SARS-CoV-2 to identify 23 nt crRNAs with a high predicted efficacy. This yielded an initial collection of 28,749 candidate targets for Cas13d. Next, Bioedit was used to identify the crRNA targets that are most conserved among different SARS-CoV-2 variants and SARS-CoV. The R script places the candidate crRNAs in four quartiles based on the predicted efficacy (Q1–Q4), with Q1 for crRNAs of modest activity and Q4 for the most active crRNAs [47]. We included crRNA candidates of all four quartiles to validate the predictive value of the R script. The selected crRNAs target important open reading frames: six non-structural proteins (nsps), five RNA-dependent RNA polymerase (RdRp; nsp12), five N protein and four viral helicase (nsp13). In addition, important replication signals were also targeted: in the leader (two), 3′-pseudoknot (three), ribosomal frameshift signal (two) and 3′-UTR (two), including the highly conserved stem-loop II motif (s2m) (Table 1, Figure 2A). In total, we selected 29 viral targets, for which crRNAs were designed to target either the +RNA or -RNA strand of the SARS-CoV-2 genome, making a total of 58 crRNAs.

### 3.2. Targeting of SARS-CoV-2 RNA by Cas13d

**Figure 3:**
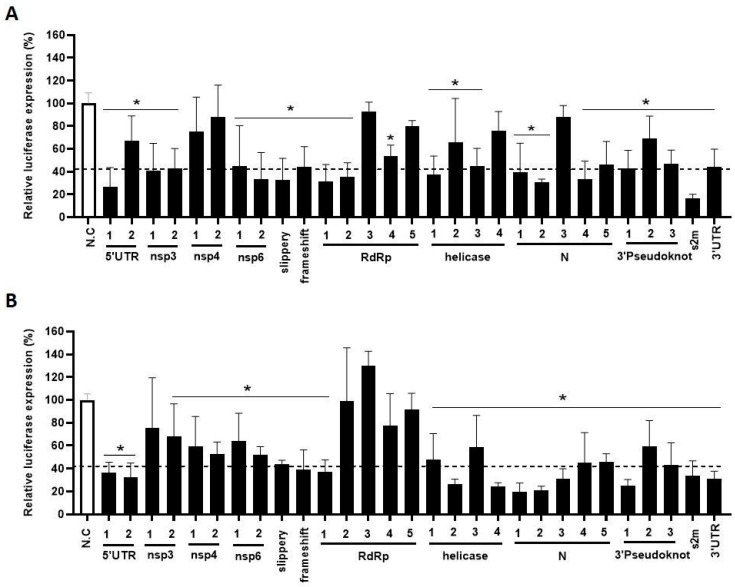
The efficiency of the designed anti-SARS-CoV-2 crRNAs. (**A**,**B**) The efficiency of 29 crRNAs targeting either the + or the -RNA strand with Cas13d was tested in HEK293T cells by co-transfecting them with the luciferase construct encoding SARS-CoV-2 +RNA (**A**) and −RNA (**B**) target sequences, respectively. To quantify viral gene expression, luciferase level was measured at two days after transfection. Mean values (±SD) of three experiments in duplicate are shown. The average luciferase levels are expressed as relative percentage level (%), setting the negative control (NC), a crRNA not targeting any SARS-CoV-2 encoding sequence, at 100%. Statistical significance was determined using one-way ANOVA, * *p* ≤ 0.05.

To test the silencing efficiency of the designed crRNAs, HEK293T cells were co-transfected with a single Cas13d-crRNA plasmid (300 ng) and one of two luciferase reporter constructs that represent either the viral + or − strand (Figure 2B). A fixed amount of Renilla plasmid was included to control for variation in the transfection efficiency. A non-targeting crRNA that targets neither SARS-CoV-2 nor human sequences was used as negative control (NC), for which the luciferase activity was set at 100%. Most crRNAs against the viral +RNA strand induced a significant reduction in the Luc signal of at least 50% (Figure 3A), but not all were active, e.g., nsp4-1, nsp4-2, RdRp-3, helicase-2 and N-3. We obtained comparable results for the set of –RNA-strand-targeting crRNAs: most crRNAs demonstrated good knockdown activity, expect for nsp3-1, RdRp-2 and RdRp-3 (Figure 3B). Overall, we obtained efficient Cas13d-mediated inhibition with the majority of the designed crRNAs, for both the + and the − strand of the SARS-CoV-2 RNA genome.

With this data set, we validated the accuracy of the in silico crRNA prediction tool of Wessels for the 29 crRNA targets in the + RNA strand. We included eight crRNAs with poor predicted activity (nsp4-1, nsp4-2, nsp6-2, N-1, RdRp-3, RdRP-4, helicase-2 and helicase-4) and the remaining 21 crRNAs with optimal predicted activity (Table 1). The experimentally determined knockdown activity did not accurately correspond to the predicted crRNA activity. For instance, N-3 was predicted to have good silencing activity but showed poor activity and nsp6-2 is a good crRNA, but was predicted to be a weak one. The correlation between the predicted guide score generated by the Wessels algorithm and the measured crRNA activity for all 29 selected target sequences was calculated, showing a significant negative correlation coefficient of −0.48 (*p* = 0.0079). Therefore, we suggest that it remains of critical importance to experimentally validate the predicted crRNA candidates.

### 3.3. A Combinatorial Antiviral crRNA Approach

**Figure 4:**
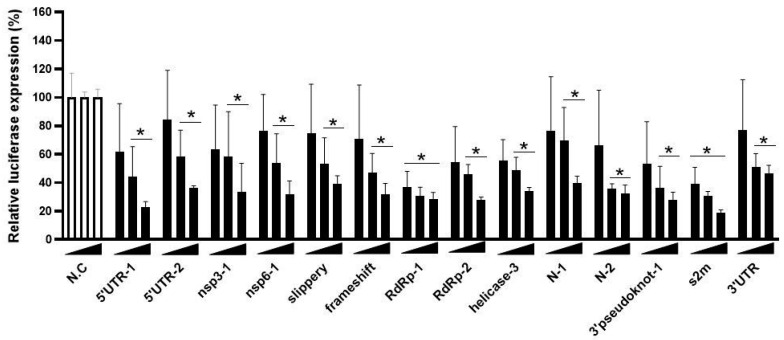
Titration of SARS-CoV-2 targeting crRNAs. The efficiency of 14 crRNAs was tested in HEK293T cells by co-transfecting them with the luciferase construct encoding SARS-CoV-2 +RNA target sequences. A titration of the crRNA constructs was performed (75, 150 and 300 ng). To quantify viral gene expression, luciferase level was measured at two days after transfection. Mean values (±SD) of three experiments in duplicate are shown. The average luciferase levels are expressed as percentage (%) of luciferase expression, setting the negative control (NC) at 100%. Statistical significance was determined using one-way ANOVA, * *p* ≤ 0.05.

We selected the 14 best (+) strand-targeting crRNAs for a dose–response test in HEK293T cells co-transfected with a fixed amount of the Luc reporter (100 ng) and increasing amounts of the Cas13d-crRNA construct: 75, 150 and 300 ng (Figure 4). These results confirmed the inhibitory potency of these crRNAs, which act in a dose-dependent manner.

**Figure 5:**
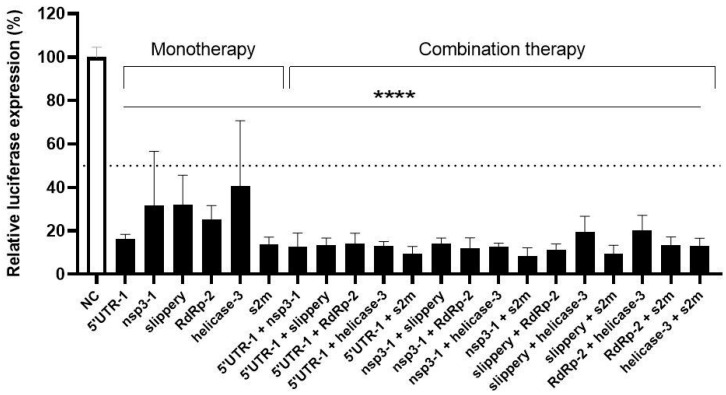
The anti-SARS-CoV-2 efficiency of single versus dual crRNA therapy. The efficiency of six crRNAs was tested in HEK293T cells by co-transfecting them with the luciferase construct encoding SARS-CoV-2 +RNA target sequences. The crRNAs were tested as single inhibitor versus a dual-combinatorial approach. To quantify viral gene expression, luciferase level was measured at two days after transfection. Mean values (±SD) of three experiments in duplicates are shown. The average luciferase levels are expressed as percentage (%) of luciferase expression, setting the negative control (NC) at 100%. Statistical significance was determined using one-way ANOVA, **** *p* ≤ 0.0001.

Like any other virus, SARS-CoV-2 will acquire spontaneous mutations in its genome during virus replication that could trigger viral escape. Viral escape will more likely occur when the crRNA inhibitor is applied as “mono-therapy”, but a combination of two or multiple crRNAs can result in additive or possibly even synergistic inhibition. A combinatorial attack will also increase the genetic threshold for the acquisition of crRNA resistance as more mutations will be required, which explains the clinical success of a combination drug therapy for HIV infection [56,57,58,59]. We selected the best crRNAs (5′UTR-1, nsp3-1, slippery, RdRp-2, helicase-3 and s2m) for a combinatorial approach and tested all possible combinations of two crRNAs in HEK293T cells co-transfected with the Luc reporter (100 ng), the Renilla plasmid (1 ng) and 300 ng of the CRISPR-Cas13d/crRNA constructs. Note that for a dual attack 150 ng of each crRNA construct was transfected, thus keeping a fixed amount of crRNA plasmid. The luciferase expression was measured at two days post-transfection and compared to the non-targeting control crRNA (NC), which was set at 100% (Figure 5). Overall, the dual crRNA combinations profoundly reduced the luciferase level to values significantly lower than those measured for the individual crRNAs. No additive effect was measured for 5′ UTR-1, possibly because it already showed strong inhibition as monotherapy.

### 3.4. The Impact of Cas13d on Intracellular SARS-CoV-2 Replication

**Figure 6:**
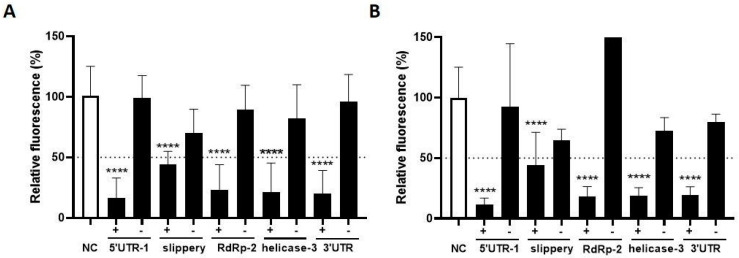
The efficiency of crRNAs targeting SARS-CoV-2 and SARS-CoV replicon. (**A**) The efficiency of five selected single crRNAs targeting SARS-CoV-2 sequences was tested in HEK293T cells by co-transfecting them with a SARS-CoV-2 replicon. crRNAs targeting both SARS-CoV-2 + and −RNA sequences were included. Viral RNA replication was quantified by measuring levels of mNeonGreen. Mean values (±SD) of three experiments in duplicates are shown. The average mNeonGreen levels are expressed as percentage (%) of fluorescent cells, setting the negative control (NC) at 100%; (**B**) The same experimental set-up was used with a SARS-CoV replicon. Here, viral RNA replication was quantified by measuring levels of GFP. Statistical significance was determined using one-way ANOVA, **** *p* ≤ 0.0001.

We next tested the inhibitory capacity of these crRNAs on SARS-CoV-2 replication using the replicon system. Replicons are sub-genomic elements that replicate the viral RNA genome inside cells without the production of infectious virus and cell-to-cell viral spread. To generate a single-cycle SARS-CoV-2 replicon, an infectious cDNA clone was assembled in a bacterial artificial chromosome (BAC) as described [49], with the structural genes replaced by the mNeonGreen reporter gene. We selected the best + and − crRNA inhibitors and tested their inhibitory capacity in HEK293T cells co-transfected with the SARS-CoV-2 replicon (Figure 6A). Two days post-transfection the mNeonGreen signal was quantitated with flow cytometry, which reflects the level of SARS-CoV-2 RNA replication. The fluorescence measured for the control crRNA (NC) was set at 100%. All five crRNAs targeting the + strand SARS-CoV-2 RNA caused a robust reduction in the mNeonGreen signal (Figure 6A). Interestingly, such suppression was not observed for four out of five crRNAs that target the -RNA strand, with the exception of the crRNA against the slippery sequence that showed modest inhibitory activity. Thus, it seems that -RNA targeting crRNAs that were fully active in the Luc assay lose their knockdown activity in the replicon assay.

Next, we evaluated the breadth of these antiviral crRNAs against the replicon of the related SARS-CoV. In this case, we actually expected optimal breadth as we selected targets that are in fact identical between SARS-CoV-2 and SARS-CoV. We measured the fluorescence signal at two days post-transfection and observed the same trend as observed for SARS-CoV-2. The crRNAs that target the +RNA strand caused a robust reduction in the fluorescent signal, demonstrating the breadth of this antiviral approach (Figure 6B). No inhibition was apparent for crRNAs that target the -RNA strand, with the exception of crRNA-slippery. Thus, we could reproduce the apparent loss of activity for most -RNA targeting crRNAs against the SARS-CoV replicon. Importantly, crRNAs that suppress the SARS-CoV-2 replicon were also able to suppress SARS-CoV, thus demonstrating the breadth of this antiviral strategy.

### 3.5. The Impact of Cas13 on the Replication Intermediate minus Sense Transcripts

**Figure 7:**
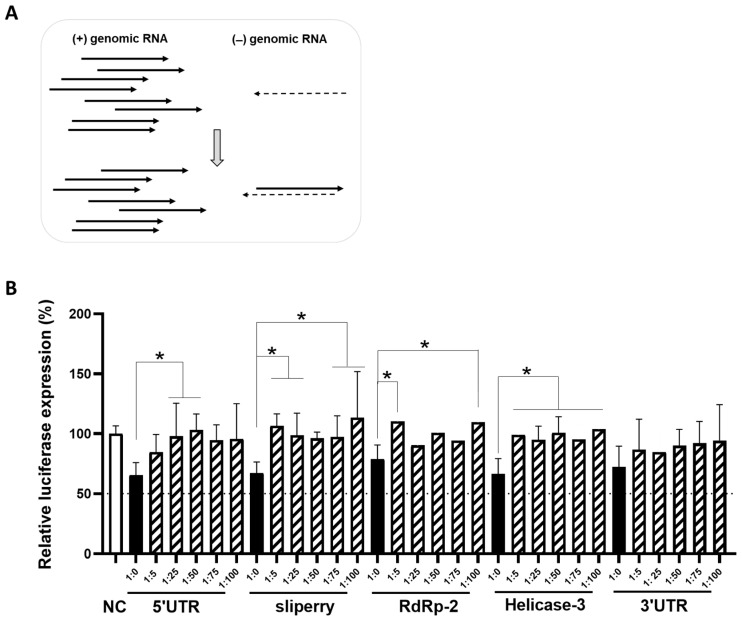
The impact of Cas13 on the intermediate minus sense transcripts. (**A**) Schematic representation of differences between +RNA and –RNA strands during SARS-CoV-2 RNA replication. +RNA strands (black arrows) are far more abudant than the –RNA strands (black dashed arrows). The less abundant –RNA strands are present as double-stranded replicative forms during RNA replication. (**B**) The efficiency of crRNAs targeting -RNA targets when annealed to complementary +RNA targets. The efficiency of six crRNAs was tested in HEK293T cells by co-transfecting them with a mixture of different ratios of − and + luciferase constructs (1:5, 1:25, 1:50, 1:75 and 1:100 of − luciferase construct: + luciferase construct) or with the − luciferase construct alone (1:0). To quantify viral gene expression, luciferase level was measured at two days after transfection. Mean values (±SD) of three experiments in duplicates are shown. The average luciferase levels are expressed as percentage (%) of luciferase expression, setting the negative control (NC) at 100%. Statistical significance was determined using one-way ANOVA, * *p* ≤ 0.05.

The lack of inhibitory activity of most crRNAs that target the -RNA strand in the replicon system is striking. To explain this, we considered the potential differences in the biology of the + and -RNA strands during coronavirus replication. A first difference is their abundance in infected cells. The -RNA strands are up to 100-fold less abundant than +RNA strands (38,69). Second, as a consequence of this difference in abundance, it is likely that most − strands will be annealed to the surplus of + strands. Such complexation is likely as both RNA strands accumulate in the same DMV compartment (69–74). DMVs are a hallmark of coronaviruses, which employ these membrane-associated structures for the replication of their RNA genome. It is possible that RNA duplex formation restricts the accessibility of all − strands for Cas13d attack, but not the surplus + strands that remain single-stranded (Figure 7A). To test this scenario, we designed a disrupted luciferase reporter with all SARS-CoV-2 + strand targets to produce a mimic of the viral + transcripts. We also made a luciferase reporter with all − targets. Both constructs were transfected in HEK293T cells with the plasmid (300 ng) expressing Cas13d and a crRNA that targets the -RNA strand. We mimicked the surplus of + strands by mixing an increasing amount of the + construct with a fixed amount of the − construct: 1:5, 1:25, 1:50, 1:75 and 1:100 (ng). We included a condition without the + construct (1:0) and compared the results to those obtained with the non-targeting crRNA (NC). We observed a gradual loss of activity of the -RNA targeting crRNA with titration of more +RNA construct (Figure 7B). Overall, this result supports the hypothesis that viral −RNA strands become inaccessible to the CRISPR-Cas13d endonuclease when forced into a stable RNA duplex by the expression of excess +RNA strands.

## 4. Discussion

The SARS-CoV-2 pandemic has triggered a search for antiviral drugs and other antiviral approaches. We show that the novel CRISPR-Cas13d endonuclease tool can be instructed for a sequence-specific attack on the viral RNA genome. We analyzed the SARS-CoV-2 genome to find the best crRNA targets of 23 nt based on our extensive expertise with sequence-specific antiviral strategies [60,61,62]. Viruses have a remarkably high mutational capacity, which creates genetic diversity that facilitates rapid adaptation to new conditions (e.g., antiviral drug pressure or a new host upon zoonotic transfer). We therefore considered viral targets of which the sequence is well-conserved among the current SARS-CoV-2 isolates. As we should prepare for a potential future outbreak of related viruses, we screened for targets that are conserved between SARS-CoV-2 and SARS-CoV. We designed a set of crRNAs to target the viral +RNA strand, but also the matching crRNAs that target the complementary -RNA strand. First, we experimentally validated the activity of these crRNAs in simple cell culture model systems with a luciferase reporter construct. The knockdown efficiency in transiently transfected cells was determined and the best crRNA antivirals were selected. This experimental validation has shown that the R script used to predict the on-target activity of crRNAs did not accurately predict the antiviral efficacy of the Cas13d-crRNAs. Therefore, experimental tests remain important in order to select the best crRNA candidates. We tested different combinations of the most potent crRNAs. The knockdown efficiency of dual crRNA combinations was significantly higher than those measured for the individual crRNAs.

Next, we used a non-infectious replicon system that contains all viral genes necessary for intracellular RNA replication and an exogenous reporter gene to quantitate the inhibition of viral RNA replication by the co-transfected crRNA constructs. CRISPR-Cas13d efficiently inhibited RNA replication when the crRNA targets the viral +RNA strand (Figure 6), but four out of five crRNAs that target the viral −RNA strand lost their inhibitory activity in the replicon assay. Consistent with these results, a recent study on antiviral siRNAs reported inactivity of those that target the viral −RNA strand [63]. We hypothesize that the differential vulnerability of viral + and −RNA strands can be explained by a profound difference in their abundance, which means that the less abundant −RNA transcripts will be annealed to the excess of viral +RNA strands inside the cells (Figure 7A). This scenario requires that both RNA transcripts be in the same location inside the infected cell, which is indeed the case as viral RNA replication occurs in DMVs in the cytoplasm. As a result of +/− duplex formation, Cas13d-mediated targeting of the viral −RNA strand is likely blocked. We mimicked this scenario with two luciferase reporters that express transcripts with a complementary sequence and demonstrated that crRNA inhibitory activity against a reporter is lost upon expression of the complementary RNA strand (Figure 7B). This result also reveals that viral RNA replication intermediates that are present inside DMVs are accessible to the Cas13d endonuclease.

Despite this dominant suppressive effect of RNA duplex formation on crRNA attack, we scored modest inhibition for the crRNA against the slippery sequence in the -RNA. This target site is unusual as it adopts a relatively stable RNA hairpin structure in the +gRNA [40,64,65] and possibly also a “mirror-image structure” in the -RNA. Such intramolecular base pairing may explain the special behavior of this target as it may interfere with intermolecular base pairing and formation of the +/−RNA duplex that facilitates crRNA annealing and Cas endonuclease cleavage. On the other hand, we observed good inhibition of several 5′/3′-UTR targets that are part of important RNA secondary structures (Figure 7A). Recent efforts to assess the influence of RNA secondary structure on Cas13 efficiency have shown a strong cleavage preference for single-stranded RNAs with 18 or more nucleotides available for base pairing to the crRNA [66]. We argue that local intramolecular base-pairing can interfere with the formation of the perfect intermolecular +/-RNA duplex and thus expose parts of the + or −RNA strand for Cas13d attack. Another factor that may influence RNA accessibility for Cas13d binding is the binding of proteins of viral or cellular origin to the viral RNA, thus forming a steric block for Cas13d annealing.

The breadth of the crRNAs was explored by testing the same crRNAs against both SARS-CoV-2 and SARS-CoV replicons. The crRNAs caused a robust reduction in the fluorescent signal, demonstrating the breadth of this antiviral approach. New SARS-CoV-2 variants will likely continue to emerge as this virus seems to be in the process of becoming endemic and there is the sustained, but low risk of yet another pandemic in the future of a “new” coronavirus. Thus, it remains of paramount importance to develop broadly active antivirals. We thus checked whether the final selection of 29 target viral sequences carry mutations in the new VOCs and confirmed that most crRNA targets are well conserved among all seven SARS-CoV-2 variants. Overall, the selected targets are conserved in at least 98% of SARS-CoV-2 variants, with the exception of 3’Pseudoknot-2 and s2m, which acquired mismatches in the Alpha and Delta variants, respectively. We have observed two insertions in the target region of 3’Pseudoknot-2 in the seed region (nucleotides 15–21) and a single point mutation for s2m. Thus, a reduced targeting efficiency of crRNA 3’Pseudoknot-2 against the Alpha variant is expected, which may not be the case for crRNA s2m with the Delta variant. The next step will therefore be to experimentally confirm the efficacy of our panel of crRNAs against these VOCs, including the highly mutated Omicron SARS-CoV-2 variant for which we found no mismatches with the designed crRNAs [67].

In conclusion, we designed and selected a panel of very active antiviral crRNAs that target highly conserved genome sequences of SARS-CoV-2 and confirmed that these antivirals also exhibit activity against the related SARS-CoV. Our crRNA design has proven to be successful as we maintain complementarity with the new SARS-CoV-2 VOCs. These results emphasize the importance of the rational design of gene-editing tools to develop antiviral strategies in order to prepare for future zoonotic outbreaks of novel pathogenic coronaviruses.

## Figures and Tables

**Table 1:** crRNAs targeting SARS-CoV-2 RNA.

Name	Nucleotide Position	crRNA Sequence	Target Sequence	Guide Score
5’UTR-1	44–66	GAGAACAGATCTACAAGAGATCG	CGATCTCTTGTAGATCTGTTCTC	0.46
5’UTR-2	53–75	GTTCGTTTAGAGAACAGATCTAC	GTAGATCTGTTCTCTAAACGAAC	0.45
nsp3-1	5666–5688	GACATCATAACAAAAGGTGACTC	GAGTCACCTTTTGTTATGATGTC	0.89
nsp3-2	7529–7551	CCATTAACAATAGTTGTACATTC	GAATGTACAACTATTGTTAATGG	0.42
nsp4-1	9584–9606	AATGTCAAGTACAAGTAAATAAC	GTTATTTACTTGTACTTGACATT	0.13
nsp4-2	9901–9923	CTCCACTAAAATACTTGTACTTA	TAAGTACAAGTATTTTAGTGGAG	0.15
nsp6-1	11603–11625	CCTAAGAAACAATAAACTAGCAT	ATGCTAGTTTATTGTTTCTTAGG	0.38
nsp6-2	11675–11697	TAAACACCAAGAGTCAGTCTAAA	TTTAGACTGACTCTTGGTGTTTA	0.24
slippery	13463–13485	ACTTACACCGCAAACCCGTTTAA	TTAAACGGGTTTGCGGTGTAAGT	0.37
frameshift	13468–13490	GCTGCACTTACACCGCAAACCCG	CGGGTTTGCGGTGTAAGTGCAGC	0.68
RdRP-1	13769–13791	TTGACGTGATATATGTGGTACCA	TGGTACCACATATATCACGTCAA	0.77
RdRP-2	14507–14529	GCTATGTAAGTTTACATCCTGAT	ATCAGGATGTAAACTTACATAGC	0.64
RdRP-3	15052–15074	TTAAGATTCATTTGAGTTATAGT	ACTATAACTCAAATGAATCTTAA	0.21
RdRP-4	15452–15474	ACCTGGTTTAACATATAGTGAAC	GTTCACTATATGTTAAACCAGGT	0.29
RdRP-5	15622–15644	CTATTTCTATAGAGACACTCATA	TATGAGTGTCTCTATAGAAATAG	0.35
Helicase-1	17011–17033	ACATTGCTAGAAAACTCATCTGA	TCAGATGAGTTTTCTAGCAATGT	0.51
Helicase-2	17221–17243	GCAGGTATAATTCTACTACATTT	AAATGTAGTAGAATTATACCTGC	0.22
Helicase-3	17479–17501	AAATATTCTGGTTCTAGTGTGCC	GGCACACTAGAACCAGAATATTT	0.44
Helicase-4	16348–16370	ACAGACAAGACTAATTTATGTGA	TCACATAAATTAGTCTTGTCTGT	0.17
N-1	28409–28431	AACCAAGACGCAGTATTATTGGG	CCCAATAATACTGCGTCTTGGTT	0.25
N-2	28434–28456	CTTGCCATGTTGAGTGAGAGCGG	CCGCTCTCACTCAACATGGCAAG	0.61
N-3	28513–28535	GGTAGTAGCCAATTTGGTCATCT	AGATGACCAAATTGGCTACTACC	0.58
N-4	28544–28566	TCACCACCACGAATTCGTCTGGT	ACCAGACGAATTCGTGGTGGTGA	0.59
N-5	29096–29118	GTTTGTTCTGGACCACGTCTGCC	GGCAGACGTGGTCCAGAACAAAC	0.51
3’Pseudoknot-1	29543–29565	TAGCCCATCTGCCTTGTGTGGTC	GACCACACAAGGCAGATGGGCTA	0.43
3’Pseudoknot-2	29586–29608	GAGTAGACTATATATCGTAAACG	CGTTTACGATATATAGTCTACTC	0.41
3’Pseudoknot-3	29638–29660	AGTTAACTACATCTACTTGTGCT	AGCACAAGTAGATGTAGTTAACT	0.38
s2m	29742–29764	CTGTACACTCGATCGTACTCCGC	GCGGAGTACGATCGAGTGTACAG	0.62
3’UTR	29787–29809	ACATTAGGGCTCTTCCATATAGG	CCTATATGGAAGAGCCCTAATGT	0.38

## Data Availability

The data underlying this article are available in the article and in its online Supplementary Material.

## Supplementary Materials

The following supporting information can be downloaded at: https://www.mdpi.com/article/10.3390/v15030686/s1. Table S1: SARS-CoV-2 genome sequences available in the GISAID database; Table S2: Shannon entropy per nucleotide position; Table S3: crRNAs oligonucleotides.
